# Computational analysis for eccentric neighborhood Zagreb indices and their significance

**DOI:** 10.1016/j.heliyon.2023.e17998

**Published:** 2023-07-11

**Authors:** Hanan Ahmed, Anwar Saleh, Rashad Ismail, Ruby Salestina M, Abdu Alameri

**Affiliations:** a Department of Mathematics, Yuvaraja's College, University of Mysore, Mysuru, India; b Department of Mathematics, Ibb University, Ibb, Yemen; c Department of Mathematics, Faculty of Science, University of Jeddah, Jeddah, Saudi Arabia; d Department of Mathematics, Faculty of Science and Arts, Mahayl Assir, King Khalid University, Saudi Arabia; e Department of Biomedical Engineering, Faculty of Engineering, University of Science and Technology, Yemen

**Keywords:** 05C12, 05C90, 05C76, Eccentric neighborhood Zagreb indices, Eccentricity neighborhood degree, Graph operations, Boiling point, Molecular descriptors

## Abstract

In this paper, a novel eccentric neighborhood degree-based topological indices, termed eccentric neighborhood Zagreb indices, have been conceptualized and its discriminating power investigated with regard to the predictability of the boiling point of the chemical substances. The discriminating power of the eccentric neighborhood Zagreb indices was compared with that of Wiener and eccentric connectivity indices. Some explicit results for those new indices for some graphs and graph operations such as join, disjunction, composition, and symmetric difference.

## 1 Introduction

A molecular graph [8], [9] is a connected simple graph such that the vertices and edges are supposed to be atoms and chemical bonds respectively. Chemical graph theory is an important branch of both chemistry and graph theory as it has taken a lot of attention because of the important results obtained in chemical graph theory and has been applied in many applications such as chemical engineering as well as pharmaceutical [13]. The main idea of chemical graph theory is that the physical and chemical properties of molecules can be studied and explained using information [10]. It can also be noted that in contemporary mathematical chemical literature, there are many descriptors of molecular structure based on vertex degree. In this research by a graph, we main undirected finite, simple and connected graph. For a graph, G=(V(G),E(G)), V(G) and E(G) denote the vertex set and edge set, respectively. The set N(u) of all neighbors of *u* is said the open neighborhood of *u*, i.e., N(u)={v∈V(G):uv∈E(G)}. The degree $d_{G}(u)=d(u)$ of a vertex *u* in *G* is defined as d(u)=|N(u)|. The length of the shortest path joining between the two vertices *u* and *v* is called the distance between those two vertices and is denoted by $d_{G}(u,v)$ or d(u,v). The origin of topological indices goes back to 1947 when a chemist by name, Wiener established the first topological index, recognize as the Wiener index [16], to search for boiling points and defined as $W(G)=\frac{1}{2}\sum_{\left\{u,v\}\in V(G\right)}d(u,v)$. Among the topological indices defined in the initial phase, Zagreb indices are related to the most common molecular descriptors. First introduced by Gutman and Trinajestic [7], the first and second Zagreb indices are given as follows:

$$M_{1}(G)=\sum_{u\in V(G)}d_{G}^{2}(u)=\sum_{uv\in E(G)}(d_{G}(u)+d_{G}(v)),$$

$$M_{2}(G)=\sum_{uv\in E(G)}d_{G}(u)d_{G}(v).$$

For more details of those indices see [3], [6], [11]. The eccentricity $\varepsilon (u)=max_{v\in V(G)}d_{G}(u,v)$.

Also, $r(G)=min_{u\in V(G)}\varepsilon (u)$ and $D(G)=max_{u\in V(G)}\varepsilon (u)$ are the radius and diameter of *G* respectively. The eccentric connectivity index [15] is defined as $\xi^{c}=\sum_{v\in V(G)}d_{G}(v)\varepsilon_{G}(v)$. For some applications of eccentric connectivity index see [5], [10], [14], and for the mathematical properties of this topological index [12], [17], [19]. The goal of this research is to define new topological indices based on new parameter known as the neighborhood eccentricity of the vertex, these new indices have a good significant to applied in chemical graph theory also have mathematical significance.

Lemma 1.1 *[4]* *Let* $G_{1}$ *and* $G_{2}$ *be any two graphs. Then*

(a) $\varepsilon_{G_{1}+\;G_{2}}(u)=\left\{ \begin{array}{cc} 1,\; & \text{if }\varepsilon_{G_{i}}(u)=1\text{;}\\ 2,\; & \text{if }\varepsilon_{G_{i}}(u)\geq 2\text{.} \end{array}\right.$
(b) $\varepsilon_{G_{1}\vee \;G_{2}}(u,v)=\left\{ \begin{array}{cc} 1,\; & \text{if }\varepsilon_{G_{1}}(u)=\varepsilon_{G_{2}}(v)=1\text{;}\\ 2,\; & \text{if }\varepsilon_{G_{1}}(u)\geq 2\text{ or }\varepsilon_{G_{2}}(v)\geq 2\text{.} \end{array}\right.$
(c) $\varepsilon_{G_{1}[G_{2}]}(u,v)=\left\{ \begin{array}{cc} 1,\; & \text{if }\varepsilon_{G_{1}}(u)=\varepsilon_{G_{2}}(v)=1\text{;}\\ 2,\; & \text{if }\varepsilon_{G_{1}}(u)=1,\;\;\varepsilon_{G_{2}}(v)\geq 2\text{;}\\ \varepsilon_{G_{1}}(u),\; & \text{if }\varepsilon_{G_{1}}(u)\geq 2\text{.} \end{array}\right.$
(d) $\varepsilon_{G_{1}⊕G_{2}}(u,v)=2$ *.*

## 2 Materials and methods

In this research, the primary amines group was adopted as a standard group in which the chemical and physical applicability of the new indices are tested. Primary amines are widely used to test the applicability of topological indices, as they were used in the Wiener index test in estimating the boiling points of these compounds [15]. Also, it is used for the structural determination of the paraffin boiling point [16]. For more studies application of topological indices on primary amines, the reader can refer to the following references [2], [18]. The values of the boiling point are described in Table 2 according to their experimental data [15], and also https://pubchem.ncbi.nlm.nih.gov After that, a non-linear regression analysis is performed using the R-program analysis, and with this analysis, the expected boiling point values of the primary amines are estimated. Linear combinations of the obtained models are plotted using Excel. This is the first part of organizing the main results of this research. In the second part, the novel-designed indices are studied and analyzed mathematically to study their properties, apply them to different families of graphs, and perform basic operations on them. We have used the analytical method, in the process.

## 3 Results and discussion

To understand the different properties of chemicals, laboratory tests must be performed, and this is extremely costly. To vanquish this problem, many topological indices in theoretical chemistry have been introduced and defined. To define a new topological index one must verify two things. The index must correspond well with at least one physical or chemical property of a standard data set, on the other hand, it should be simple in the formulation it and give some theoretical insight. In this section, we have two subsections. First, we define the significance of the first, second, and third eccentric neighborhood Zagreb indices in determining the predicted boiling point using nonlinear regression analysis. Second, we study the eccentric neighborhood Zagreb indices mathematically.

### 3.1 The significance of the eccentric neighborhood Zagreb indices in predicting the boiling point of molecular descriptors

**Figure 1:**
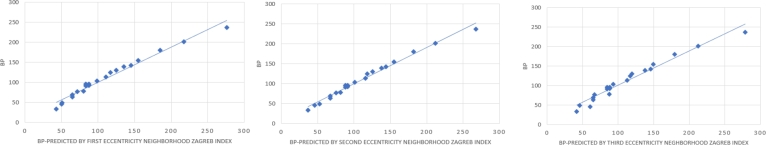
Linear fitting of BP predicted by (a) *E*_*N*_*M*_1_(*G*), (b) *E*_*N*_*M*_2_(*G*), (c) *E*_*N*_*M*_3_(*G*) with BP.

**Figure 2:**
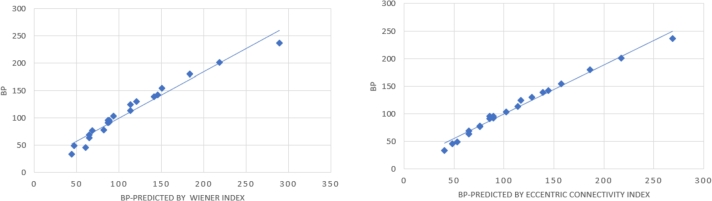
Linear fitting of BP predicted by (a) *W*(*G*) with BP, (b) *ξ*^*c*^(*G*) with BP.

To verify the importance and the efficiency of a topological index for modeling physicalchemical properties we use nonlinear regression analysis. Commonly, for such an investigation, primary amines are useful because of their diverse structurally. In this section, we find the Wiener index and eccentric connectivity index with the eccentric neighborhood Zagreb indices and the data listed in Table 1. We get the relationship of eccentric neighborhood Zagreb indices with boiling points of primary amines as in Table 2. Table 3, is shown that the predicted boiling points calculated by the first, second, and third eccentric neighborhood Zagreb indices are strongly correlated with boiling points of primary amines (R=0.987), (R=0.993) and (R=0.9814) respectively, (see Fig. 1, a, b and c). Also, we present the correlation coefficient of boiling points predicted by the eccentric connectivity index and Wiener index with these indices (see Fig. 2, a and b). In Table 4, we determined the correlation coefficient of $E_{N}M_{1}(G)$, $E_{N}M_{2}(G)$ and $E_{N}M_{3}(G)$ with $\xi^{c}(G)$ and W(G). For more delicate statistical tests, which rank the indices by their predictive power, see Table 5.

**Table 1:** Eccentric neighborhood Zagreb indices with eccentric connectivity index and Wiener index of primary amines.

	Compound	*ξ*^*c*^(*G*)	*W*(*G*)	*E*_*N*_*M*_1_(*G*)	*E*_*N*_*M*_2_(*G*)	*E*_*N*_*M*_3_(*G*)
1	n-propylamine	14	10	58	45	24
2	2-aminopropane	9	9	39	18	21
3	2-amino-2-methylpropane	12	16	56	32	36
4	2-aminobutane	19	18	101	82	40
5	2-methylpropylamine	19	18	101	82	40
6	n-butylamine	24	20	126	108	42
7	2-amino-2-methylbutane	24	28	162	131	62
8	2-aminopentane	31	32	199	174	63
9	3-methylbutylamine	31	31	199	174	63
10	2-methylbutylamine	29	32	175	162	59
11	n-pentylamine	38	35	258	225	68
12	4-methylpentylamine	47	50	379	332	95
13	n-hexylamine	54	56	442	388	98
14	3-methylpentylamine	45	50	339	318	90
15	4-aminoheptane	61	75	531	488	121
16	2-aminoheptane	65	79	623	546	131
17	n-heptylamine	74	84	722	654	136
18	n-octylamine	96	120	1078	972	178
19	n-nonylamine	122	165	1562	1425	228
20	2-aminoundecane	169	275	2687	2474	339
21	3-aminopentane	29	31	175	162	59

**Table 2:** Relationship of predicted boiling points calculated by eccentric neighborhood Zagreb indices, *ξ*^*c*^(*G*) and *W*(*G*) with BP of primary amines.

Compound	BP	BP *E*_*N*_*M*_1_(*G*)	BP *E*_*N*_*M*_2_(*G*)	BP *E*_*N*_*M*_3_(*G*)	BP *ξ*^*c*^(*G*)	BP *W*(*G*)
n-propylamine	49	50.94	52.67	45.63	53.27	46.7
2-aminopropane	33	42.76	36.41	41.65	39.97	44.1
2-amino-2-methylpropane	46	50.12	45.91	60.19	48.19	60.52
2-aminobutane	63	65.06	67.08	64.68	64.97	64.57
2-methylpropylamine	69	65.06	67.08	64.68	64.97	64.57
n-butylamine	77	71.72	74.96	66.87	75.6	68.42
2-amino-2-methylbutane	78	80.13	81.02	87.25	75.62	82.33
2-aminopentane	92	87.74	90.84	88.21	89.31	88.6
3-methylbutylamine	96	87.74	90.84	88.21	89.31	87.07
2-methylbutylamine	96	82.9	88.26	84.35	85.52	88.6
n-pentylamine	104	98.38	100.75	92.94	101.94	93.08
4-methylpentylamine	125	116.57	117.86	116.78	117.05	113.25
n-hexylamine	130	124.74	125.49	119.29	128.1	120.54
3-methylpentylamine	114	110.97	115.83	112.55	113.79	113.25
4-aminoheptane	139	135.26	137.65	137.76	138.67	141.55
2-aminoheptane	142	145.13	144.02	145.44	144.5	145.65
n-heptylamine	155	154.88	154.88	149.21	157.22	150.65
n-octylamine	180	184.83	181.7	179.32	186.2	183.3
n-nonylamine	201	217.67	211.99	212.35	217.59	218.39
2-aminoundecane	237	276.5	267.77	278.43	268.93	289.23
3-aminopentane	91	82.9	88.26	84.35	85.52	87.07

**Table 3:** Correlation coefficient of boiling points predicted by eccentric neighborhood Zagreb indices, *ξ*^*c*^(*G*) and *W*(*G*) with BP of primary amines.

	BP *E*_*N*_*M*_1_(*G*)	BP *E*_*N*_*M*_2_(*G*)	BP *E*_*N*_*M*_3_(*G*)	BP *ξ*^*c*^(*G*)	BP *W*(*G*)
BP	0.987	0.993	0.9814	0.99199	0.97875

**Table 4:** Correlation coefficients of *E*_*N*_*M*_1_(*G*), *E*_*N*_*M*_2_(*G*) and *E*_*N*_*M*_3_(*G*) with *ξ*^*c*^(*G*) and *W*(*G*).

	*E*_*N*_*M*_1_(*G*)	*E*_*N*_*M*_2_(*G*)	*E*_*N*_*M*_3_(*G*)	*ξ*^*c*^(*G*)	*W*(*G*)
*E*_*N*_*M*_1_(*G*)	1				
*E*_*N*_*M*_2_(*G*)	0.9999	1			
*E*_*N*_*M*_3_(*G*)	0.9869	0.9865	1		
*ξ*^*c*^(*G*)	0.9807	0.9802	0.9962	1	
*W*(*G*)	0.9978	0.9979	0.994	0.9872	1

**Table 5:** Some delicate statistical tests, which rank the indices by their predictive power.

	Residual Standard Error on 19 degree of freedom	Multiple R-Squared	Adjusted R-Squared	F-Statistic on 1 and 19 DF	P-Value
*E*_*N*_*M*_1_(*G*)	0.09442	0.966	0.9642	539.2	2.077 × 10^−15^
*E*_*N*_*M*_2_(*G*)	0.05331	0.9891	0.9886	1732	<2.2 × 10^−16^
$E_{N}M_{1}^{⁎}(G)$	0.1161	0.9485	0.9458	350.1	1.064 × 10^−13^
*ξ*^*c*^(*G*)	0.07367	0.9793	0.9782	897.9	<2.2 × 10^−16^
*W*(*G*)	0.1209	0.9442	0.9413	321.4	2.301 × 10^−13^

The non linear regression analysis equations which are used are:

$$ln(BP)=2.14+0.441\;\;ln(E_{N}M_{1}(G)),$$

$$ln(BP)=2.43+0.403\;\;ln(E_{N}M_{2}(G)),$$

$$ln(BP)=1.65+0.683\;\;ln(E_{N}M_{3}(G)),$$

$$ln(BP)=2.26+0.65\;\;ln(\xi^{c}(G)),$$

ln(BP)=2.578+0.55ln(W(G)).

### 3.2 Eccentric neighborhood Zagreb indices of graphs and graph operations

Definition 3.1 Let G=(V(G),E(G)) be a connected simple graph and $\delta_{en}(v)=\sum_{u\in N(v)}\varepsilon (u)$ be the eccentricity neighborhood degree. Then the first, second and third eccentric neighborhood Zagreb indices are defined as follows:

$$E_{N}M_{1}(G)=\sum_{v\in V(G)}\delta_{en}^{2}(v),$$

$$E_{N}M_{2}(G)=\sum_{uv\in E(G)}\delta_{en}(u)\delta_{en}(v),$$

$$E_{N}M_{3}(G)=\sum_{uv\in E(G)}(\delta_{en}(u)+\delta_{en}(v)).$$

Proposition 3.2

1. *For star graph*$S_{r}$*with*r+1*vertices, we have*$E_{N}M_{1}(S_{r})=4r^{2}+r$*,*$E_{N}M_{2}(S_{r})=2r^{2}$*and*$E_{N}M_{3}(S_{r})=2r^{2}+r$*.*
2. *If*$G≅S_{r,s}$*with*r+s+2*vertices, then*$E_{N}M_{1}(G)=9{\left(r^{2}+s^{2}\right)}^{2}+16(r+s)+8$*,*$E_{N}M_{2}(G)=(2+3r)(2r+3s+2)+2s(2+3s)$*and*$E_{N}M_{3}(G)=(4+3r)r+(3s+4)s+3(r+s)+4$*.*
3. *Suppose*$G≅C_{n}$*,*n≥4*. Then*$E_{N}M_{1}(G)=E_{N}M_{2}(G)=\left\{ \begin{array}{cc} n^{3},\; & \text{if n is even;}\\ n{\left(n-1\right)}^{2},\; & \text{if n is odd.} \end{array}\right.$
  $E_{N}M_{3}(G)=\left\{ \begin{array}{cc} 2n^{2},\; & \text{if n is even;}\\ 2n(n-1),\; & \text{if n is odd.} \end{array}\right.$
4. *If*$G≅W_{n}$*is the wheel graph with*n≥5*vertices, then*$E_{N}M_{1}(W_{n})=4n^{2}+17n-21$*,*$E_{N}M_{2}(W_{n})=10n^{2}+5n-15$*, and*$E_{N}M_{3}(W_{n})=2n^{2}+11n-13$*.*

Lemma 3.3 *Let*$G≅P_{n}$*with vertex set*$\left\{u_{1},u_{2},...,u_{n}\right\}$*. Then*

1. *If n is even and*n≥4*, then*$\delta_{en}(u_{i})=\left\{ \begin{array}{cc} n-2,\; & \text{if }i=1,n\text{;}\\ 2n-2i,\; & \text{if }i=2,3,...,(\frac{n}{2}-1)\text{;}\\ 2i-2,\; & \text{if }i=(\frac{n}{2}+2),...,n-1\text{;}\\ n+1,\; & \text{if }i=\frac{n}{2},(\frac{n}{2}+1)\text{.} \end{array}\right.$
2. *If n is odd and*n≥3*, then*$\delta_{en}(u_{i})=\left\{ \begin{array}{cc} n-2,\; & \text{if }i=1,n\text{;}\\ 2n-2i,\; & \text{if }i=2,3,...,(\frac{n+1}{2}-1)\text{;}\\ 2i-2,\; & \text{if }i=(\frac{n+1}{2}+1),...,n-1\text{;}\\ n+1,\; & \text{if }i=\frac{n+1}{2}\text{.} \end{array}\right.$

Proposition 3.4

1. *Let*$P_{n}$*be a path with*n≥4*where n is even, then*
  $$E_{N}M_{1}(P_{n})=\frac{1}{3}(7n^{3}-27n^{2}+38n+6),\;\;\;\;E_{N}M_{3}(P_{n})=3n^{2}-8n+8.$$
2. *If*n≥6*is even, then*
  $$E_{N}M_{2}(P_{n})=\frac{1}{3}(7n^{3}-33n^{2}+56n+15).$$
3. *Let*$P_{n}$*be a path with*n≥3*where n is odd, then*
  $$E_{N}M_{1}(P_{n})=\frac{1}{3}(7n^{3}-27n^{2}+35n+3),\;\;\;\;E_{N}M_{3}(P_{n})=3n^{2}-8n+7.$$
4. *If n is odd and*n≥5*, then*
  $$E_{N}M_{2}(P_{n})=\frac{1}{3}(7n^{3}-33n^{2}+53n+9).$$

An (r,s) banana tree denoted by $B_{r,s}$, defined by Chan et al. [1], is a graph obtained by connecting one leaf of each of *r* copies of an *s*-star graph with a single root vertex that is different from all the stars.

Lemma 3.5 *If*$G≅B_{r,s}$*with*r≥2*, and*s≥3*and w is the root vertex, then*

$\delta_{en}(v)=\left\{ \begin{array}{cc} 5,\; & \text{if v is pendent vertex;}\\ 4r,\; & \text{if v=w;}\\ 6s-8,\; & \text{if v is the center vertex;}\\ 8,\; & \text{if v }\in \{v:vw\in E(B_{r,s})\}\text{.} \end{array}\right.$

Proposition 3.6 *Suppose*$G≅B_{r,s}$*with*r≥2*and*s≥3*. Then*

$$E_{N}M_{1}(G)=16r^{2}+36s^{2}r-71sr+78r,$$

$$E_{N}M_{2}(G)=32r^{2}+30s^{2}r-52sr+16r,$$

$$E_{N}M_{3}(G)=4r^{2}+6s^{2}r-9rs+14r.$$

Proposition 3.7 *For any graph G*

$$\sum_{v\in V(G)}\delta_{en}(v)=\xi^{c}(G).$$

Proposition 3.8 *Suppose G is a graph with diameter and radius*D(G)*and*r(G)*respectively, then*

$r^{2}(G)M_{1}(G)\leq E_{N}M_{1}(G)\leq D^{2}(G)M_{1}(G)$*,*
$r^{2}(G)M_{2}(G)\leq E_{N}M_{2}(G)\leq D^{2}(G)M_{2}(G)$*,*
$r(G)M_{1}(G)\leq E_{N}M_{3}(G)\leq D(G)M_{1}(G)$*.*

*Equality holds if and only if*D(G)=1*.*

### 3.3 Join

A join [11] G+H of two graphs *G* and *H* with V(G) and V(H) as disjoint vertex sets is the graph on the vertex set V(G)∪V(H) and the edge set $E(G)\cup E(H)\cup \{u_{1}u_{2}:u_{1}\in V(G),u_{2}\in V(H)\}$.

Lemma 3.9 *For any two graphs G and H*

(a) *If*$V_{\omega }(G)=V_{\omega }(H)=\emptyset$*, then*$\delta_{en_{G+H}}(u)=2d_{G+H}(u)$*.*
(b) *If*$|V_{\omega }(G)|=r$*and*$|V_{\omega }(H)|=s$*such that,*r+s>0*, then*
  $\delta_{en_{G+H}}(u)=\left\{ \begin{array}{cc} 2d_{G}(u)+2|V(H)|-(r+s),\; & \text{if }u\in V(G),u\notin V_{\omega }(G)\text{;}\\ 2d_{G}(u)+2|V(H)|+1-(r+s),\; & \text{if }u\in V(G),u\in V_{\omega }(G)\text{;}\\ 2d_{H}(u)+2|V(G)|-(r+s),\; & \text{if }u\in V(H),u\notin V_{\omega }(H)\text{;}\\ 2d_{H}(u)+2|V(G)|+1-(r+s),\; & \text{if }u\in V(H),u\in V_{\omega }(H)\text{.} \end{array}\right.$

We can partition the edge set of E(G+H) as follows:

$$E_{1}^{1}=\{uv\in E(G):u,v\notin V_{w}(G)\},E_{1}^{2}=\{uv\in E(G):u,v\in V_{w}(G)\},$$

$$E_{1}^{3}=\{uv\in E(G):u\notin V_{w}(G),\;v\in V_{w}(G)\},E_{2}^{1}=\{uv\in E(H):u,v\notin V_{w}(H)\},$$

$$E_{2}^{2}=\{uv\in E(H):u,v\in V_{w}(H)\},\;E_{2}^{3}=\{uv\in E(H):u\notin V_{w}(H),\;v\in V_{w}(H)\}.$$

Let $E_{u\in V(G),\;v\in V(H)}$ be the set of edges connecting vertices of *G* with vertices of *H*. Then

$$E_{3}^{1}=\{uv\in E_{u\in V(G),\;v\in V(H)}:u\notin V_{w}(G),v\notin V_{w}(H)\},$$

$$E_{3}^{2}=\{uv\in E_{u\in V(G),\;v\in V(H)}:u\notin V_{w}(G),v\in V_{w}(H)\},$$

$$E_{3}^{3}=\{uv\in E_{u\in V(G),\;v\in V(H)}:u\in V_{w}(G),v\notin V_{w}(H)\},$$

$$E_{3}^{4}=\{uv\in E_{u\in V(G),\;v\in V(H)}:u\in V_{w}(G),v\in V_{w}(H)\}.$$

Theorem 3.10 *Let G and H be any two graphs with*$|V_{\omega }(G)|=r$*,*$|V_{\omega }(H)|=s$*and*r+s>0*. Then*

$$\left(a)\;\;E_{N}M_{1}(G+H)=4M_{1}(G+H)-8(r+s)(|E(G)|+|E(H)|)+4(\sum_{\begin{array}{c} u\in V(G)\\ u\in V_{\omega }(G) \end{array}}d_{G}(u)+\sum_{\begin{array}{c} u\in V(H)\\ u\in V_{\omega }(H) \end{array}}d_{H}(u))+(r+s)({\left(r+s\right)}^{2}-2(r+s)-4(r|V(H)|+s|V(G)|))+(r+s)[(r+s-4|V(H)|)(|V(G)|-1)+(r+s-4|V(G)|)(|V(H)|-s)]+(4|V(H)|+1)r+(4|V(G)|+1)s.(b)\;\;E_{N}M_{2}(G+H)=4(M_{2}(G)+M_{2}(H)+|V(H)|M_{1}(G)+|V(G)|M_{1}(H))+4(\sum_{\begin{array}{c} u\in V(G)\\ v\in V(H) \end{array}}(d_{G}(u)d_{H}(v))+\sum_{\begin{array}{c} u\in V(G)\\ v\in V(H) \end{array}}(|V(G)|d_{G}(u)+|V(H)|d_{H}(v)))-2(r+s)(\sum_{uv\in E_{1}^{1}}(d_{G}(u)+d_{G}(v))+\sum_{uv\in E_{2}^{1}}(d_{H}(u)+d_{H}(v))+\sum_{uv\in E_{3}^{1}}(d_{G}(u)+d_{H}(v)))-2(r+s-1)(\sum_{uv\in E_{1}^{2}}(d_{G}(u)+d_{G}(v))+\sum_{uv\in E_{2}^{2}}(d_{H}(u)+d_{H}(v))+\sum_{uv\in E_{3}^{4}}(d_{G}(u)+d_{H}(v))\right)$$

$$-2[\sum_{\begin{array}{c} uv\in E_{1}^{3}\\ u\notin V_{\omega }(G)\\ v\in V_{\omega }(G) \end{array}}((r+s-1)d_{G}(u)+(r+s)d_{G}(v))+\sum_{\begin{array}{c} uv\in E_{2}^{3}\\ u\notin V_{\omega }(H)\\ v\in V_{\omega }(H) \end{array}}((r+s-1)d_{H}(u)+(r+s)d_{H}(v))+\sum_{\begin{array}{c} uv\in E_{3}^{2}\\ u\notin V_{\omega }(G)\\ v\in V_{\omega }(H) \end{array}}((r+s-1)d_{G}(u)+(r+s)d_{H}(v))+\sum_{\begin{array}{c} uv\in E_{3}^{3}\\ u\in V_{\omega }(G)\\ v\notin V_{\omega }(H) \end{array}}((r+s)d_{G}(u)+(r+s-1)d_{H}(v))]+{\left(2|V(H)|-(r+s)\right)}^{2}|E_{1}^{1}|+{\left(2|V(H)|-(r+s-1)\right)}^{2}|E_{1}^{2}|+(4|V(H){|}^{2}-2|V(H)|(2(r+s)-1)+(r+s)(r+s-1))|E_{1}^{3}|+{\left(2|V(G)|-(r+s)\right)}^{2}|E_{2}^{1}|+{\left(2|V(G)|-(r+s-1)\right)}^{2}|E_{2}^{2}|+(4|V(G){|}^{2}-2|V(G)|(2(r+s)-1)+(r+s)(r+s-1))|E_{2}^{3}|+(4|V(G)||V(H)|-2(r+s)(|V(G)|+|V(H)|)+{\left(r+s\right)}^{2})|E_{3}^{1}|+(4|V(G)||V(H)|-2(|V(G)|(r+s)+|V(H)|(r+s-1))+(r+s)(r+s-1))|E_{3}^{2}|+(4|V(G)||V(H)|-2(|V(H)|(r+s)+|V(G)|(r+s-1))+(r+s)(r+s-1))|E_{3}^{3}|+(4|V(G)||V(H)|-2(r+s-1)(|V(G)|+|V(H)|)+{\left(r+s-1\right)}^{2})|E_{3}^{4}|.$$

$$(c)\;\;E_{N}M_{3}(G+H)=2(M_{1}(G)+M_{1}(H))+(4|V(H)|-2(r+s))|E(G)|+(4|V(G)|-2(r+s))|E(H)|+2(|E_{1}^{2}|+|E_{2}^{2}|)+|E_{1}^{3}|+|E_{2}^{3}|+2\sum_{\begin{array}{c} u\in V(G)\\ v\in V(H) \end{array}}(d_{G}(u)+d_{H}(v))+(2(|V(G)|+|V(H)|)-2(r+s))|V(G)||V(H)|+|E_{3}^{2}|+|E_{3}^{3}|+2|E_{3}^{4}|.$$

Proof Applying Lemma 3.9(b), we get

$$E_{N}M_{1}(G+H)=\sum_{u\in V(G+H)}\delta_{en_{G+H}}^{2}(u)=\sum_{\begin{array}{c} u\in V(G)\\ u\notin V_{\omega }(G) \end{array}}\delta_{en_{G+H}}^{2}(u)+\sum_{\begin{array}{c} u\in V(G)\\ u\in V_{\omega }(G) \end{array}}\delta_{en_{G+H}}^{2}(u)+\sum_{\begin{array}{c} u\in V(H)\\ u\notin V_{\omega }(H) \end{array}}\delta_{en_{G+H}}^{2}(u)+\sum_{\begin{array}{c} u\in V(H)\\ u\in V_{\omega }(H) \end{array}}\delta_{en_{G+H}}^{2}(u)=\sum_{\begin{array}{c} u\in V(G)\\ u\notin V_{\omega }(G) \end{array}}{\left(2d_{G}(u)+2|V(H)|-(r+s)\right)}^{2}+\sum_{\begin{array}{c} u\in V(G)\\ u\in V_{\omega }(G) \end{array}}{\left(2d_{G}(u)+2|V(H)|+1-(r+s)\right)}^{2}+\sum_{\begin{array}{c} u\in V(H)\\ u\notin V_{\omega }(H) \end{array}}{\left(2d_{H}(u)+2|V(G)|-(r+s)\right)}^{2}+\sum_{\begin{array}{c} u\in V(H)\\ u\in V_{\omega }(H) \end{array}}(2d_{H}(u)+2|V(G)|{+1-(r+s))}^{2}=4\sum_{\begin{array}{c} u\in V(G)\\ u\notin V_{\omega }(G) \end{array}}{\left(d_{G}(u)+|V(H)|\right)}^{2}-4(r+s)\sum_{\begin{array}{c} u\in V(G)\\ u\notin V_{\omega }(G) \end{array}}d_{G}(u)+({\left(r+s\right)}^{2}-4|V(H)|(r+s))(|V(G)|-r)+4\sum_{\begin{array}{c} u\in V(G)\\ u\in V_{\omega }(G) \end{array}}{\left(d_{G}(u)+|V(H)|\right)}^{2}+4(1-(r+s))\sum_{\begin{array}{c} u\in V(G)\\ u\in V_{\omega }(G) \end{array}}d_{G}(u)+(4|V(H)|+1)r+((r+s)-2-4|V(H)|)(r+s)r+4\sum_{\begin{array}{c} u\in V(H)\\ u\notin V_{\omega }(H) \end{array}}{\left(d_{H}(u)+|V(G)|\right)}^{2}-4(r+s)\sum_{\begin{array}{c} u\in V(H)\\ u\notin V_{\omega }(H) \end{array}}d_{H}(u)+({\left(r+s\right)}^{2}-4|V(G)|(r+s))(|V(H)|-s)+4\sum_{\begin{array}{c} u\in V(H)\\ u\in V_{\omega }(H) \end{array}}{\left(d_{H}(u)+|V(G)|\right)}^{2}+4(1-(r+s))\sum_{\begin{array}{c} u\in V(H)\\ u\in V_{\omega }(H) \end{array}}d_{H}(u)+(4|V(G)|+1)s+(r+s-2-4|V(G)|)(r+s)s=4M_{1}(G+H)-8(r+s)(|E(G)|+|E(H)|)+4(\sum_{\begin{array}{c} u\in V(G)\\ u\in V_{\omega }(G) \end{array}}d_{G}(u)+\sum_{\begin{array}{c} u\in V(H)\\ u\in V_{\omega }(H) \end{array}}d_{H}(u))$$

$$+(r+s)({\left(r+s\right)}^{2}-2(r+s)-4(r|V(H)|+s|V(G)|))+(r+s)[(r+s-4|V(H)|)(|V(G)|-1)+(r+s-4|V(G)|)(|V(H)|-s)]+(4|V(H)|+1)r+(4|V(G)|+1)s.$$

To prove (b) and (c) we use the partition of the edge set E(G+H) as mentioned earlier. Hence applying Lemma 3.9(b), we get

$$E_{N}M_{2}(G+H)=\sum_{uv\in E(G+H)}\delta_{en_{G+H}}(u)\delta_{en_{G+H}}(v)=\sum_{uv\in E_{1}^{1}}\delta_{en_{G+H}}(u)\delta_{en_{G+H}}(v)+\sum_{uv\in E_{1}^{2}}\delta_{en_{G+H}}(u)\delta_{en_{G+H}}(v)+\sum_{\begin{array}{c} uv\in E_{1}^{3}\\ u\notin V_{\omega }(G)\\ v\in V_{\omega }(G) \end{array}}\delta_{en_{G+H}}(u)\delta_{en_{G+H}}(v)+\sum_{uv\in E_{2}^{1}}\delta_{en_{G+H}}(u)\delta_{en_{G+H}}(v)+\sum_{uv\in E_{2}^{2}}\delta_{en_{G+H}}(u)\delta_{en_{G+H}}(v)+\sum_{\begin{array}{c} uv\in E_{2}^{3}\\ u\notin V_{\omega }(H)\\ v\in V_{\omega }(H) \end{array}}\delta_{en_{G+H}}(u)\delta_{en_{G+H}}(v)+\sum_{uv\in E_{3}^{1}}\delta_{en_{G+H}}(u)\delta_{en_{G+H}}(v)+\sum_{\begin{array}{c} uv\in E_{3}^{2}\\ u\notin V_{\omega }(G)\\ v\in V_{\omega }(H) \end{array}}\delta_{en_{G+H}}(u)\delta_{en_{G+H}}(v)+\sum_{\begin{array}{c} uv\in E_{3}^{3}\\ u\in V_{\omega }(G)\\ v\notin V_{\omega }(H) \end{array}}\delta_{en_{G+H}}(u)\delta_{en_{G+H}}(v)+\sum_{uv\in E_{3}^{4}}\delta_{en_{G+H}}(u)\delta_{en_{G+H}}(v)=\sum_{uv\in E_{1}^{1}}((2d_{G}(u)+2|V(H)|-(r+s))(2d_{G}(v)+2|V(H)|-(r+s)))+\sum_{\begin{array}{c} uv\in E_{1}^{2} \end{array}}((2d_{G}(u)+2|V(H)|+1-(r+s))(2d_{G}(v)+2|V(H)|+1-(r+s)))+\sum_{\begin{array}{c} uv\in E_{1}^{3}\\ u\notin V_{\omega }(G)\\ v\in V_{\omega }(G) \end{array}}((2d_{G}(u)+2|V(H)|-(r+s))(2d_{G}(v)+2|V(H)|+1-(r+s)))+\sum_{uv\in E_{2}^{1}}((2d_{H}(u)+2|V(G)|-(r+s))(2d_{H}(v)$$

$$+2|V(G)|-(r+s)))+\sum_{uv\in E_{2}^{2}}((2d_{H}(u)+2|V(G)|+1-(r+s))(2d_{H}(v)+2|V(G)|+1-(r+s)))+\sum_{\begin{array}{c} uv\in E_{2}^{3}\\ u\notin V_{\omega }(H)\\ v\in V_{\omega }(H) \end{array}}((2d_{H}(u)+2|V(G)|-(r+s))(2d_{H}(v)+2|V(G)|+1-(r+s)))+\sum_{uv\in E_{3}^{1}}((2d_{G}(u)+2|V(H)|-(r+s))(2d_{H}(v)+2|V(G)|-(r+s)))+\sum_{\begin{array}{c} uv\in E_{3}^{2}\\ u\notin V_{\omega }(G)\\ v\in V_{\omega }(H) \end{array}}((2d_{G}(u)+2|V(H)|-(r+s))(2d_{H}(v)+2|V(G)|+1-(r+s)))+\sum_{\begin{array}{c} uv\in E_{3}^{3}\\ u\in V_{\omega }(G)\\ v\notin V_{\omega }(H) \end{array}}((2d_{G}(u)+2|V(H)|+1-(r+s))(2d_{H}(v)+2|V(G)|-(r+s)))+\sum_{uv\in E_{3}^{4}}((2d_{G}(u)+2|V(H)|+1-(r+s))(2d_{H}(v)+2|V(G)|+1-(r+s)))=4M_{2}(G)+(4|V(H)|-2(r+s))\sum_{uv\in E_{1}^{1}}(d_{G}(u)+d_{G}(v))+{\left(2|V(H)|-(r+s)\right)}^{2}|E_{1}^{1}|+(4|V(H)|-2(r+s-1))\sum_{uv\in E_{1}^{2}}(d_{G}(u)+d_{G}(v))+{\left(2|V(H)|-(r+s-1)\right)}^{2}|E_{1}^{2}|+4|V(H)|\sum_{\begin{array}{c} uv\in E_{1}^{3}\\ u\notin V_{\omega }(G)\\ v\in V_{\omega }(G) \end{array}}(d_{G}(u)+d_{G}(v))-2\sum_{\begin{array}{c} uv\in E_{1}^{3}\\ u\notin V_{\omega }(G)\\ v\in V_{\omega }(G) \end{array}}((r+s-1)d_{G}(u)+(r+s)d_{G}(v))+[4|V(H){|}^{2}-2|V(H)|(2(r+s)-1)+(r+s)(r+s-1)]|E_{1}^{3}|+4M_{2}(H)+(4|V(G)|-2(r+s))\sum_{uv\in E_{2}^{1}}(d_{H}(u)+d_{H}(v))+{\left(2|V(G)|-(r+s)\right)}^{2}|E_{2}^{1}|+(4|V(G)|-2(r+s-1))\sum_{uv\in E_{2}^{2}}(d_{H}(u)+d_{H}(v))+{\left(2|V(G)|-(r+s-1)\right)}^{2}|E_{2}^{2}|+4|V(G)|\sum_{\begin{array}{c} uv\in E_{2}^{3}\\ u\notin V_{\omega }(H)\\ v\in V_{\omega }(H) \end{array}}(d_{H}(u)+d_{H}(v))-2\sum_{\begin{array}{c} uv\in E_{2}^{3}\\ u\notin V_{\omega }(H)\\ v\in V_{\omega }(H) \end{array}}((r+s-1)d_{H}(u)+(r+s)d_{H}(v))+[4|V(G){|}^{2}-2|V(G)|(2(r+s)-1)+(r+s)(r+s-1)]|E_{2}^{3}|+4\sum_{\begin{array}{c} u\in V(G)\\ v\in V(H) \end{array}}(d_{G}(u)d_{H}(v))-2(r+s)(\sum_{uv\in E_{3}^{1}}(d_{G}(u)+d_{H}(v))+\sum_{uv\in E_{3}^{4}}(d_{G}(u)+d_{H}(v)))$$

$$+2\sum_{uv\in E_{3}^{4}}(d_{G}(u)+d_{H}(v))+4[\sum_{uv\in E_{3}^{1}}(|V(G)|d_{G}(u)+|V(H)|d_{H}(v))+\sum_{\begin{array}{c} uv\in E_{3}^{2}\\ u\notin V_{\omega }(G)\\ v\in V_{\omega }(H) \end{array}}(|V(G)|d_{G}(u)+|V(H)|d_{H}(v))+\sum_{\begin{array}{c} uv\in E_{3}^{3}\\ u\in V_{\omega }(G)\\ v\notin V_{\omega }(H) \end{array}}(|V(G)|d_{G}(u)+|V(H)|d_{H}(v))+\sum_{uv\in E_{3}^{4}}(|V(G)|d_{G}(u)+|V(H)|d_{H}(v))]-2(\sum_{\begin{array}{c} uv\in E_{3}^{2}\\ u\notin V_{\omega }(G)\\ v\in V_{\omega }(H) \end{array}}((r+s-1)d_{G}(u)+(r+s)d_{H}(v))+\sum_{\begin{array}{c} uv\in E_{3}^{3}\\ u\in V_{\omega }(G)\\ v\notin V_{\omega }(H) \end{array}}((r+s)d_{G}(u)+(r+s-1)d_{H}(v)))+[4|V(G)||V(H)|-2(r+s)(|V(G)|+|V(H)|)+{\left(r+s\right)}^{2}]|E_{3}^{1}|+[4|V(G)||V(H)|-2(|V(G)|(r+s)+|V(H)|(r+s-1))+(r+s)(r+s-1)]|E_{3}^{2}|+[4|V(G)||V(H)|-2(|V(H)|(r+s)+|V(G)|(r+s-1))+(r+s)(r+s-1)]|E_{3}^{3}|+[4|V(G)||V(H)|-2(r+s-1)(|V(G)|+|V(H)|)+{\left(r+s-1\right)}^{2}]|E_{3}^{4}|=4(M_{2}(G)+M_{2}(H)+|V(H)|M_{1}(G)+|V(G)|M_{1}(H))+4(\sum_{\begin{array}{c} u\in V(G)\\ v\in V(H) \end{array}}(d_{G}(u)d_{H}(v))+\sum_{\begin{array}{c} u\in V(G)\\ v\in V(H) \end{array}}(|V(G)|d_{G}(u)+|V(H)|d_{H}(v)))-2(r+s)(\sum_{uv\in E_{1}^{1}}(d_{G}(u)+d_{G}(v))+\sum_{uv\in E_{2}^{1}}(d_{H}(u)+d_{H}(v))+\sum_{uv\in E_{3}^{1}}(d_{G}(u)+d_{H}(v)))-2(r+s-1)(\sum_{\begin{array}{c} uv\in E_{1}^{2} \end{array}}(d_{G}(u)+d_{G}(v))+\sum_{uv\in E_{2}^{2}}(d_{H}(u)+d_{H}(v))+\sum_{uv\in E_{3}^{4}}(d_{G}(u)+d_{H}(v)))-2[\sum_{\begin{array}{c} uv\in E_{1}^{3}\\ u\notin V_{\omega }(G)\\ v\in V_{\omega }(G) \end{array}}((r+s-1)d_{G}(u)+(r+s)d_{G}(v))+\sum_{\begin{array}{c} uv\in E_{2}^{3}\\ u\notin V_{\omega }(H)\\ v\in V_{\omega }(H) \end{array}}((r+s-1)d_{H}(u)+(r+s)d_{H}(v))+\sum_{\begin{array}{c} uv\in E_{3}^{2}\\ u\notin V_{\omega }(G)\\ v\in V_{\omega }(H) \end{array}}((r+s-1)d_{G}(u)+(r+s)d_{H}(v))$$

$$+\sum_{\begin{array}{c} uv\in E_{3}^{3}\\ u\in V_{\omega }(G)\\ v\notin V_{\omega }(H) \end{array}}((r+s)d_{G}(u)+(r+s-1)d_{H}(v))]+{\left(2|V(H)|-(r+s)\right)}^{2}|E_{1}^{1}|+{\left(2|V(H)|-(r+s-1)\right)}^{2}|E_{1}^{2}|+(4|V(H){|}^{2}-2|V(H)|(2(r+s)-1)+(r+s)(r+s-1))|E_{1}^{3}|+{\left(2|V(G)|-(r+s)\right)}^{2}|E_{2}^{1}|+{\left(2|V(G)|-(r+s-1)\right)}^{2}|E_{2}^{2}|+(4|V(G){|}^{2}-2|V(G)|(2(r+s)-1)+(r+s)(r+s-1))|E_{2}^{3}|+(4|V(G)||V(H)|-2(r+s)(|V(G)|+|V(H)|)+{\left(r+s\right)}^{2})|E_{3}^{1}|+(4|V(G)||V(H)|-2(|V(G)|(r+s)+|V(H)|(r+s-1))+(r+s)(r+s-1))|E_{3}^{2}|+(4|V(G)||V(H)|-2(|V(H)|(r+s)+|V(G)|(r+s-1))+(r+s)(r+s-1))|E_{3}^{3}|+(4|V(G)||V(H)|-2(r+s-1)(|V(G)|+|V(H)|)+{\left(r+s-1\right)}^{2})|E_{3}^{4}|.$$

$$E_{N}M_{3}(G+H)=\sum_{uv\in E(G+H)}(\delta_{en_{G+H}}(u)+\delta_{en_{G+H}}(v))=\sum_{uv\in E_{1}^{1}}(\delta_{en_{G+H}}(u)+\delta_{en_{G+H}}(v))+\sum_{uv\in E_{1}^{2}}(\delta_{en_{G+H}}(u)+\delta_{en_{G+H}}(v))+\sum_{\begin{array}{c} uv\in E_{1}^{3}\\ u\notin V_{\omega }(G)\\ v\in V_{\omega }(G) \end{array}}(\delta_{en_{G+H}}(u)+\delta_{en_{G+H}}(v))+\sum_{uv\in E_{2}^{1}}(\delta_{en_{G+H}}(u)+\delta_{en_{G+H}}(v))+\sum_{uv\in E_{2}^{2}}(\delta_{en_{G+H}}(u)+\delta_{en_{G+H}}(v))+\sum_{\begin{array}{c} uv\in E_{2}^{3}\\ u\notin V_{\omega }(H)\\ v\in V_{\omega }(H) \end{array}}(\delta_{en_{G+H}}(u)+\delta_{en_{G+H}}(v))+\sum_{uv\in E_{3}^{1}}(\delta_{en_{G+H}}(u)+\delta_{en_{G+H}}(v))+\sum_{\begin{array}{c} uv\in E_{3}^{2}\\ u\notin V_{\omega }(G)\\ v\in V_{\omega }(H) \end{array}}(\delta_{en_{G+H}}(u)+\delta_{en_{G+H}}(v))+\sum_{\begin{array}{c} uv\in E_{3}^{3}\\ u\in V_{\omega }(G)\\ v\notin V_{\omega }(H) \end{array}}(\delta_{en_{G+H}}(u)+\delta_{en_{G+H}}(v))+\sum_{uv\in E_{3}^{4}}(\delta_{en_{G+H}}(u)+\delta_{en_{G+H}}(v))$$

$$=\sum_{uv\in E_{1}^{1}}((2d_{G}(u)+2|V(H)|-(r+s))+(2d_{G}(v)+2|V(H)|-(r+s)))+\sum_{\begin{array}{c} uv\in E_{1}^{2} \end{array}}((2d_{G}(u)+2|V(H)|+1-(r+s))+(2d_{G}(v)+2|V(H)|+1-(r+s)))+\sum_{\begin{array}{c} uv\in E_{1}^{3}\\ u\notin V_{\omega }(G)\\ v\in V_{\omega }(G) \end{array}}((2d_{G}(u)+2|V(H)|-(r+s))+(2d_{G}(v)+2|V(H)|+1-(r+s)))+\sum_{uv\in E_{2}^{1}}((2d_{H}(u)+2|V(G)|-(r+s))+(2d_{H}(v)+2|V(G)|-(r+s)))+\sum_{uv\in E_{2}^{2}}((2d_{H}(u)+2|V(G)|+1-(r+s))+(2d_{H}(v)+2|V(G)|+1-(r+s)))+\sum_{\begin{array}{c} uv\in E_{2}^{3}\\ u\notin V_{\omega }(H)\\ v\in V_{\omega }(H) \end{array}}((2d_{H}(u)+2|V(G)|-(r+s))+(2d_{H}(v)+2|V(G)|+1-(r+s)))+\sum_{uv\in E_{3}^{1}}((2d_{G}(u)+2|V(H)|-(r+s))+(2d_{H}(v)+2|V(G)|-(r+s)))+\sum_{\begin{array}{c} uv\in E_{3}^{2}\\ u\notin V_{\omega }(G),\;\;v\in V_{\omega }(H) \end{array}}((2d_{G}(u)+2|V(H)|-(r+s))+(2d_{H}(v)+2|V(G)|+1-(r+s)))+\sum_{\begin{array}{c} uv\in E_{3}^{3}\\ u\in V_{\omega }(G),v\notin V_{\omega }(H) \end{array}}((2d_{G}(u)+2|V(H)|+1-(r+s))+(2d_{H}(v)+2|V(G)|-(r+s)))+\sum_{uv\in E_{3}^{4}}((2d_{G}(u)+2|V(H)|+1-(r+s))+(2d_{H}(v)+2|V(G)|+1-(r+s)))=2M_{1}(G)+(4|V(H)|-2(r+s))|E_{1}^{1}(G)|+(4|V(H)|+2-2(r+s))|E_{1}^{2}(G)|+(4|V(H)|+1-2(r+s))|E_{1}^{3}(G)|+2M_{1}(H)+(4|V(G)|-2(r+s))|E_{2}^{1}(H)|+(4|V(G)|+2-2(r+s))|E_{2}^{2}(H)|+(4|V(G)|+1-2(r+s))|E_{2}^{3}(H)|+2\sum_{\begin{array}{c} u\in V(G)\\ v\in V(H) \end{array}}(d_{G}(u)+d_{H}(v))+(2(|V(G)|+|V(H)|)+1-2(r+s))(|E_{3}^{2}|+|E_{3}^{3}|)+(2(|V(G)|+|V(H)|)-2(r+s))|E_{3}^{1}|+(2(|V(G)|+|V(H)|)+2-2(r+s))|E_{3}^{4}|$$

$$=2(M_{1}(G)+M_{1}(H))+(4|V(H)|-2(r+s))|E(G)|+(4|V(G)|-2(r+s))|E(H)|+2(|E_{1}^{2}|+|E_{2}^{2}|)+|E_{1}^{3}|+|E_{2}^{3}|+2\sum_{\begin{array}{c} u\in V(G)\\ v\in V(H) \end{array}}(d_{G}(u)+d_{H}(v))+(2(|V(G)|+|V(H)|)-2(r+s))|V(G)||V(H)|+|E_{3}^{2}|+|E_{3}^{3}|+2|E_{3}^{4}|.$$

 □

Corollary 3.11 *Suppose G and H are any two graphs such that,*$V_{\omega }(G)=V_{\omega }(H)=\emptyset$*. Then*

$$(a)\;\;\;E_{N}M_{1}(G+H)=4M_{1}(G+H),\;\;\;E_{N}M_{3}(G+H)=2M_{1}(G+H),$$

$$E_{N}M_{2}(G+H)=4M_{2}(G+H).$$

$$(b)\;\;\;E_{N}M_{3}(G+H)=2(M_{1}(G)+M_{1}(H))+4(|V(H)||E(G)|+|V(G)|+|E(H)|)+|V(H)|(4|E(G)|+2|V(H)||V(G)|)+|V(G)|(4|E(H)|+2|V(H)||V(G)|).$$

Example 3.12 $E_{N}M_{1}(K_{r,s})=4(rs^{2}+sr^{2})$, $E_{N}M_{2}(K_{r,s})=4r^{2}s^{2}$ and $E_{N}M_{3}(K_{r,s})=2(rs^{2}+sr^{2})$.

Example 3.13 For n≥4, we have

$$E_{N}M_{1}(P_{2}+P_{n})=8n^{2}+44n-38,$$

$$E_{N}M_{2}(P_{2}+P_{n})=28n^{2}+36n-67,$$

$$E_{N}M_{3}(P_{2}+P_{n})=4n^{2}+30n-22.$$

### 3.4 Disjunction

The disjunction G∨H [11] is the graph with vertex set V(G)×V(H) in which (x,y) is adjacent with (z,w) whenever xz∈E(G) or yw∈E(H).

Lemma 3.14 *Suppose G and H are two graphs. Then*

(a) *If G and H are complete graphs, then*$\delta_{en\;G\vee H}(u,v)=d_{G\vee H}(u,v)$*.*
(b) *If*$V_{\omega }(G)=\emptyset$*or*$V_{\omega }(H)=\emptyset$*, then*$\delta_{en_{G\vee H}}(u,v)=2d_{G\vee H}(u,v)$*.*
(c) *If*$V_{\omega }(G)$*and*$V_{\omega }(H)$*are not empty sets, such that,*$|V_{\omega }(G)|=r$*,*$|V_{\omega }(H)|=s$*, then*
  $\delta_{en_{G\vee H}}(u,v)=\left\{ \begin{array}{cc} 2d_{G\vee H}(u,v)+1-rs,\; & \text{if }\varepsilon_{G\vee H}(u,v)=1\text{;}\\ 2d_{G\vee H}(u,v)-rs,\; & \text{otherwise.} \end{array}\right.$

One can partition the edges of E(G∨H) as: $E_{1}$ be the set of edges connecting the vertices which satisfy $\varepsilon_{G\vee H}(u,v)=1$, $E_{2}$ be the set of edges connecting the vertices which satisfy $\varepsilon_{G\vee H}(u,v)\neq 1$ and $E_{3}$ be the set of edges connecting the vertices which satisfy $\varepsilon_{G\vee H}(u,v)=1$ with the vertices which satisfy $\varepsilon_{G\vee H}(u,v)\neq 1$.

Theorem 3.15 *Suppose G and H are two graphs such that,*$|V_{\omega }(G)|=r$*,*$|V_{\omega }(H)|=s$*with*rs>0*. Then*

$$(a)\;\;E_{N}M_{1}(G\vee H)=4M_{1}(G\vee H)-8rs|E(G\vee H)|+(1-2rs+rs|V(G\vee H)|)rs+4\sum_{\begin{array}{c} \left(u,v)\in V(G\vee H\right)\\ \varepsilon_{G\vee H}(u,v)=1 \end{array}}d_{G\vee H}(u,v).$$

$$(b)\;\;E_{N}M_{2}(G\vee H)=4M_{2}(G\vee H)-2((rs-1)\sum_{((a,b),(c,d))\in E_{1}}(d_{G\vee H}(a,b)+d_{G\vee H}(c,d))+rs\sum_{((a,b),(c,d))\in E_{2}}(d_{G\vee H}(a,b)+d_{G\vee H}(c,d))+\sum_{\begin{array}{c} ((a,b),(c,d))\in E_{3}\\ \varepsilon_{G\vee H}(a,b)=1,\\ \varepsilon_{G\vee H}(c,d)\neq 1 \end{array}}((rs)d_{G\vee H}(a,b)+(rs-1)d_{G\vee H}(c,d)))+{\left(rs-1\right)}^{2}|E_{1}|+rs(rs|E_{2}|+(rs-1)|E_{3}|).$$

$$(c)\;\;E_{N}M_{3}(G\vee H)=2M_{1}(G\vee H)-2rs|E(G\vee H)|+2|E_{1}|+|E_{3}|.$$

Proof (a) Applying Lemma 3.14 (c), we have

$$E_{N}M_{1}(G\vee H)=\sum_{\left(u,v)\in V(G\vee H\right)}\delta_{en_{G\vee H}}^{2}(u,v)=\sum_{\begin{array}{c} \left(u,v)\in V(G\vee H\right)\\ \varepsilon_{G\vee H}(u,v)=1 \end{array}}\delta_{en_{G\vee H}}^{2}(u,v)+\sum_{\begin{array}{c} \left(u,v)\in V(G\vee H\right)\\ \varepsilon_{G\vee H}(u,v)=2 \end{array}}\delta_{en_{G\vee H}}^{2}(u,v)=\sum_{\begin{array}{c} \left(u,v)\in V(G\vee H\right)\\ \varepsilon_{G\vee H}(u,v)=1 \end{array}}{\left(2d_{G\vee H}(u,v)+1-rs\right)}^{2}+\sum_{\begin{array}{c} \left(u,v)\in V(G\vee H\right)\\ \varepsilon_{G\vee H}(u,v)=2 \end{array}}{\left(2d_{G\vee H}(u,v)-rs\right)}^{2}$$

$$=4\sum_{\begin{array}{c} \left(u,v)\in V(G\vee H\right)\\ \varepsilon_{G\vee H}(u,v)=1 \end{array}}d_{G\vee H}^{2}(u,v)+4(1-rs)\sum_{\begin{array}{c} \left(u,v)\in V(G\vee H\right)\\ \varepsilon_{G\vee H}(u,v)=1 \end{array}}d_{G\vee H}(u,v)+(1-2rs+r^{2}s^{2})rs+4\sum_{\begin{array}{c} \left(u,v)\in V(G\vee H\right)\\ \varepsilon_{G\vee H}(u,v)=2 \end{array}}d_{G\vee H}^{2}(u,v)-4rs\sum_{\begin{array}{c} \left(u,v)\in V(G\vee H\right)\\ \varepsilon_{G\vee H}(u,v)=2 \end{array}}d_{G\vee H}(u,v)+r^{2}s^{2}(|V(G\vee H)|-rs)=4M_{1}(G\vee H)-8rs|E(G\vee H)|+(1-2rs+rs|V(G\vee H)|)rs+4\sum_{\begin{array}{c} \left(u,v)\in V(G\vee H\right)\\ \varepsilon_{G\vee H}(u,v)=1 \end{array}}d_{G\vee H}(u,v).$$

To prove (b) and (c) we use the edge partition of E(G∨H) as mentioned earlier. Hence by Lemma 3.14(c), we have

$$\left(b)\;\;E_{N}M_{2}(G\vee H)=\sum_{\left((a,b),(c,d))\in E(G\vee H\right)}\delta_{en_{G\vee H}}(a,b)\delta_{en_{G\vee H}}(c,d)=\sum_{((a,b),(c,d))\in E_{1}}\delta_{en_{G\vee H}}(a,b)\delta_{en_{G\vee H}}(c,d)+\sum_{((a,b),(c,d))\in E_{2}}\delta_{en_{G\vee H}}(a,b)\delta_{en_{G\vee H}}(c,d)+\sum_{\begin{array}{c} ((a,b),(c,d))\in E_{3}\\ \varepsilon_{G\vee H}(a,b)=1\\ \varepsilon_{G\vee H}(c,d)\neq 1 \end{array}}\delta_{en_{G\vee H}}(a,b)\delta_{en_{G\vee H}}(c,d)=\sum_{((a,b),(c,d))\in E_{1}}(2d_{G\vee H}(a,b)+1-rs)(2d_{G\vee H}(c,d)+1-rs)+\sum_{((a,b),(c,d))\in E_{2}}(2d_{G\vee H}(a,b)-rs)(2d_{G\vee H}(c,d)-rs)+\sum_{\begin{array}{c} ((a,b),(c,d))\in E_{3}\\ \varepsilon_{G\vee H}(a,b)=1\\ \varepsilon_{G\vee H}(c,d)\neq 1 \end{array}}(2d_{G\vee H}(a,b)-(rs-1))(2d_{G\vee H}(c,d)-rs)=4\sum_{((a,b),(c,d))\in E_{1}}d_{G\vee H}(a,b)d_{G\vee H}(c,d)+2(1-rs)\sum_{((a,b),(c,d))\in E_{1}}(d_{G\vee H}(a,b)+d_{G\vee H}(c,d))+{\left(1-rs\right)}^{2}|E_{1}\right|$$

$$+4\sum_{((a,b),(c,d))\in E_{2}}d_{G\vee H}(a,b)d_{G\vee H}(c,d)-2rs\sum_{((a,b),(c,d))\in E_{2}}(d_{G\vee H}(a,b)+d_{G\vee H}(c,d))+r^{2}s^{2}|E_{2}|+4\sum_{\begin{array}{c} ((a,b),(c,d))\in E_{3}\\ \varepsilon_{G\vee H}(a,b)=1\\ \varepsilon_{G\vee H}(c,d)\neq 1 \end{array}}d_{G\vee H}(a,b)d_{G\vee H}(c,d)-2\sum_{\begin{array}{c} ((a,b),(c,d))\in E_{3}\\ \varepsilon_{G\vee H}(a,b)=1\\ \varepsilon_{G\vee H}(c,d)\neq 1 \end{array}}(rs\;d_{G\vee H}(a,b)+(rs-1)\;d_{G\vee H}(c,d))+rs(rs-1)|E_{3}|=4M_{2}(G\vee H)-2((rs-1)\sum_{((a,b),(c,d))\in E_{1}}(d_{G\vee H}(a,b)+d_{G\vee H}(c,d))+rs\sum_{((a,b),(c,d))\in E_{2}}(d_{G\vee H}(a,b)+d_{G\vee H}(c,d))+\sum_{\begin{array}{c} ((a,b),(c,d))\in E_{3}\\ \varepsilon_{G\vee H}(a,b)=1\\ \varepsilon_{G\vee H}(c,d)\neq 1 \end{array}}((rs)d_{G\vee H}(a,b)+(rs-1)d_{G\vee H}(c,d)))+{\left(rs-1\right)}^{2}|E_{1}|+rs(rs|E_{2}|+(rs-1)|E_{3}|).$$

$$(c)\;\;E_{N}M_{3}(G\vee H)=\sum_{\left((a,b),(c,d))\in E(G\vee H\right)}\delta_{en_{G\vee H}}(a,b)+\delta_{en_{G\vee H}}(c,d)=\sum_{((a,b),(c,d))\in E_{1}}\delta_{en_{G\vee H}}(a,b)+\delta_{en_{G\vee H}}(c,d)+\sum_{((a,b),(c,d))\in E_{2}}\delta_{en_{G\vee H}}(a,b)+\delta_{en_{G\vee H}}(c,d)+\sum_{\begin{array}{c} ((a,b),(c,d))\in E_{3}\\ \varepsilon_{G\vee H}(a,b)=1\\ \varepsilon_{G\vee H}(c,d)\neq 1 \end{array}}\delta_{en_{G\vee H}}(a,b)+\delta_{en_{G\vee H}}(c,d)=2M_{1}(G\vee H)-2rs|E(G\vee H)|+2|E_{1}|+|E_{3}|.$$

 □

Corollary 3.16

(a) *If*$V_{\omega }(G)=\emptyset \;\;or\;\;\;V_{\omega }(H)=\emptyset$*, then*
  $$E_{N}M_{1}(G\vee H)=4M_{1}(G\vee H),\;\;\;E_{N}M_{2}(G\vee H)=4M_{2}(G\vee H),$$
  $$E_{N}M_{3}(G\vee H)=2M_{1}(G\vee H).$$
(b) *If G and H are two complete graphs, then*
  $$E_{N}M_{1}(G\vee H)=E_{N}M_{3}(G\vee H)=M_{1}(G\vee H),\;\;\;E_{N}M_{2}(G\vee H)=M_{2}(G\vee H).$$

Example 3.17 For n≥4, we have

$$E_{N}M_{1}(P_{2}\vee P_{n})=2n^{3}+8n^{2}-12,$$

$$E_{N}M_{2}(P_{2}\vee P_{n})=4n^{4}+24n^{3}+24n^{2}-48n-48,$$

$$E_{N}M_{3}(P_{2}\vee P_{n})=8n^{3}+32n^{2}-48.$$

### 3.5 Composition

The composition [11] of *G* and *H* having V(G) and V(H) as vertex sets and E(G) and E(H) as edge sets is a graph G[H] containing vertex set V(G)×V(H) and (a,b) is connected to (c,d) if and only if ac∈E(G) or a=c and bd∈E(H).

Lemma 3.18 *For any two graphs G and H*

(a) *If G and H are complete graphs, then*$\delta_{en_{G[H]}}(u,v)=d_{G[H]}(u,v)$*.*
(b) *If G has at least one vertex with*ε(u)=1*and H does not have any vertex with*ε(u)=1*, then*$\delta_{en_{G[H]}}(u,v)=2d_{G[H]}(u,v)$*.*
(c) *If*$V_{\omega }(G)$*and*$V_{\omega }(H)$*are not empty sets, such that,*$|V_{\omega }(G)|=r$*,*$|V_{\omega }(H)|=s$*, then*
  $\delta_{en_{G[H]}}(u,v)=\left\{ \begin{array}{cc} 2d_{G[H]}(u,v)+1-rs,\; & \text{if }\varepsilon_{G[H]}(u,v)=1\text{;}\\ 2d_{G[H]}(u,v)-rs,\; & \text{otherwise.} \end{array}\right.$

Theorem 3.19 *For any two graphs G and H with*$|V_{\omega }(G)|=r$*,*$|V_{\omega }(H)|=s$*and*rs>0*. Then*

$$(a)\;\;E_{N}M_{1}(G[H])=4M_{1}(G[H])-8rs|E(G[H])|+4\sum_{\begin{array}{c} \left(u,v)\in V(G[H]\right)\\ \varepsilon_{G[H]}(u,v)=1 \end{array}}d_{G[H]}(u,v)+(1-2rs+rs|V(G[H])|)rs.$$

$$(b)\;\;E_{N}M_{2}(G[H])=4M_{2}(G[H])-2[(rs-1)\sum_{((a,b),(c,d))\in E_{1}}(d_{G[H]}(a,b)+d_{G[H]}(c,d))+rs\sum_{((a,b),(c,d))\in E_{2}}(d_{G[H]}(a,b)+d_{G[H]}(c,d))+\sum_{\begin{array}{c} ((a,b),(c,d))\in E_{3}\\ \varepsilon_{G[H]}(a,b)=1\\ \varepsilon_{G[H]}(c,d)\neq 1 \end{array}}((rs)d_{G[H]}(a,b)+(rs-1)d_{G[H]}(c,d))]+{\left(rs-1\right)}^{2}|E_{1}|+rs(rs|E_{2}|+(rs-1)|E_{3}|).$$

$$(c)\;\;E_{N}M_{3}(G[H])=2M_{1}(G[H])-2rs|E(G[H])|+2|E_{1}|+|E_{3}|.$$

Proof (a) Applying Lemma 3.18(c), we get

$$E_{N}M_{1}(G[H])=\sum_{\left(u,v)\in V(G[H]\right)}\delta_{en_{G[H]}}^{2}(u,v)=\sum_{\begin{array}{c} \left(u,v)\in V(G[H]\right)\\ \varepsilon_{G[H]}(u,v)=1 \end{array}}\delta_{en_{G[H]}}^{2}(u,v)+\sum_{\begin{array}{c} \left(u,v)\in V(G[H]\right)\\ \varepsilon_{G[H]}(u,v)\neq 1 \end{array}}\delta_{en_{G[H]}}^{2}(u,v)=\sum_{\begin{array}{c} \left(u,v)\in V(G[H]\right)\\ \varepsilon_{G[H]}(u,v)=1 \end{array}}{\left(2d_{G[H]}(u,v)-(rs-1)\right)}^{2}+\sum_{\begin{array}{c} \left(u,v)\in V(G[H]\right)\\ \varepsilon_{G[H]}(u,v)\neq 1 \end{array}}{\left(2d_{G[H]}-rs\right)}^{2}=4\sum_{\begin{array}{c} \left(u,v)\in V(G[H]\right)\\ \varepsilon_{G[H]}(u,v)=1 \end{array}}d_{G[H]}^{2}(u,v)-4(rs-1)\sum_{\begin{array}{c} \left(u,v)\in V(G[H]\right)\\ \varepsilon_{G[H]}(u,v)=1 \end{array}}d_{G[H]}(u,v)+{\left(rs-1\right)}^{2}rs+4\sum_{\begin{array}{c} \left(u,v)\in V(G[H]\right)\\ \varepsilon_{G[H]}(u,v)\neq 1 \end{array}}d_{G[H]}^{2}(u,v)-4rs\sum_{\begin{array}{c} \left(u,v)\in V(G[H]\right)\\ \varepsilon_{G[H]}(u,v)\neq 1 \end{array}}d_{G[H]}(u,v)+r^{2}s^{2}|V(G[H])-rs|=4M_{1}(G[H])-8rs|E(G[H])|+4\sum_{\begin{array}{c} \left(u,v)\in V(G[H]\right)\\ \varepsilon_{G[H]}(u,v)=1 \end{array}}d_{G[H]}(u,v)+(1-2rs+rs|V(G[H]))rs.$$

To prove (b) and (c) we use the edge partition of E(G[H]) which is similar to the edge partition of E(G∨H). Hence by Lemma 3.18(c), we have

$$\left(b)\;\;E_{N}M_{2}(G[H])=\sum_{\left((a,b),(c,d))\in E(G[H]\right)}\delta_{en_{G[H]}}(a,b)\delta_{en_{G[H]}}(c,d)=\sum_{((a,b),(c,d))\in E_{1}}\delta_{en_{G[H]}}(a,b)\delta_{en_{G[H]}}(c,d)+\sum_{((a,b),(c,d))\in E_{2}}\delta_{en_{G[H]}}(a,b)\delta_{en_{G[H]}}(c,d\right)$$

$$+\sum_{\begin{array}{c} ((a,b),(c,d))\in E_{3}\\ \varepsilon_{G[H]}(a,b)=1\\ \varepsilon_{G[H]}(c,d)\neq 1 \end{array}}\delta_{en_{G[H]}}(a,b)\delta_{en_{G[H]}}(c,d)=\sum_{((a,b),(c,d))\in E_{1}}(2d_{G[H]}(a,b)-(rs-1))(2d_{G[H]}(c,d)-(rs-1))+\sum_{((a,b),(c,d))\in E_{2}}(2d_{G[H]}(a,b)-rs)(2d_{G[H]}(c,d)-rs)+\sum_{\begin{array}{c} ((a,b),(c,d))\in E_{3}\\ \varepsilon_{G[H]}(a,b)=1\\ \varepsilon_{G[H]}(c,d)\neq 1 \end{array}}(2d_{G[H]}(a,b)-(rs-1))(2d_{G[H]}(c,d)-rs)=4\sum_{((a,b),(c,d))\in E_{1}}d_{G[H]}(a,b)d_{G[H]}(c,d)-2(rs-1)\sum_{((a,b),(c,d))\in E_{1}}(d_{G[H]}(a,b)+d_{G[H]}(c,d))+{\left(rs-1\right)}^{2}|E_{1}|+4\sum_{((a,b),(c,d))\in E_{2}}d_{G[H]}(a,b)d_{G[H]}(c,d)-2rs\sum_{((a,b),(c,d))\in E_{2}}(d_{G[H]}(a,b)+d_{G[H]}(c,d))+r^{2}s^{2}|E_{2}|+4\sum_{\begin{array}{c} ((a,b),(c,d))\in E_{3}\\ \varepsilon_{G[H]}(a,b)=1\\ \varepsilon_{G[H]}(c,d)\neq 1 \end{array}}d_{G[H]}(a,b)d_{G[H]}(c,d)-2\sum_{\begin{array}{c} ((a,b),(c,d))\in E_{3}\\ \varepsilon_{G[H]}(a,b)=1\\ \varepsilon_{G[H]}(c,d)\neq 1 \end{array}}(rsd_{G[H]}(a,b)+(rs-1)d_{G[H]}(c,d))+rs(rs-1)|E_{3}|=4M_{2}(G[H])-2[(rs-1)\sum_{((a,b),(c,d))\in E_{1}}(d_{G[H]}(a,b)+d_{G[H]}(c,d))+rs\sum_{((a,b),(c,d))\in E_{2}}(d_{G[H]}(a,b)+d_{G[H]}(c,d))+\sum_{\begin{array}{c} ((a,b),(c,d))\in E_{3}\\ \varepsilon_{G[H]}(a,b)=1\\ \varepsilon_{G[H]}(c,d)\neq 1 \end{array}}((rs)d_{G[H]}(a,b)+(rs-1)d_{G[H]}(c,d))]+{\left(rs-1\right)}^{2}|E_{1}|+rs(rs|E_{2}|+(rs-1)|E_{3}|).$$

$$(c)\;\;E_{N}M_{3}(G[H])=\sum_{\left((a,b),(c,d))\in E(G[H]\right)}\delta_{en_{G[H]}}(a,b)+\delta_{en_{G[H]}}(c,d)=\sum_{((a,b),(c,d))\in E_{1}}\delta_{en_{G[H]}}(a,b)+\delta_{en_{G[H]}}(c,d)+\sum_{((a,b),(c,d))\in E_{2}}\delta_{en_{G[H]}}(a,b)+\delta_{en_{G[H]}}(c,d)+\sum_{\begin{array}{c} ((a,b),(c,d))\in E_{3}\\ \varepsilon_{G[H]}(a,b)=1\\ \varepsilon_{G[H]}(c,d)\neq 1 \end{array}}\delta_{en_{G[H]}}(a,b)+\delta_{en_{G[H]}}(c,d)=\sum_{((a,b),(c,d))\in E_{1}}((2d_{G[H]}(a,b)-(rs-1))+(2d_{G[H]}(c,d)-(rs-1)))+\sum_{((a,b),(c,d))\in E_{2}}((2d_{G[H]}(a,b)-rs)+(2d_{G[H]}(c,d)-rs))+\sum_{\begin{array}{c} ((a,b),(c,d))\in E_{3}\\ \varepsilon_{G[H]}(a,b)=1\\ \varepsilon_{G[H]}(c,d)\neq 1 \end{array}}((2d_{G[H]}(a,b)-(rs-1))+(2d_{G[H]}(c,d)-rs))=2\sum_{((a,b),(c,d))\in E_{1}}(d_{G[H]}(a,b)+d_{G[H]}(c,d))-2(rs-1)|E_{1}|+2\sum_{((a,b),(c,d))\in E_{2}}(d_{G[H]}(a,b)+d_{G[H]}(c,d))-2rs|E_{2}|+\sum_{\begin{array}{c} ((a,b),(c,d))\in E_{3}\\ \varepsilon_{G[H]}(a,b)=1\\ \varepsilon_{G[H]}(c,d)\neq 1 \end{array}}(d_{G[H]}(a,b)+d_{G[H]}(c,d))-(2rs-1)|E_{3}|=2M_{1}(G[H])-2rs|E(G[H])|+2|E_{1}|+|E_{3}|.$$

 □

Corollary 3.20 *If G and H are complete graphs, then*$E_{N}M_{1}(G[H])=E_{N}M_{3}(G[H])=M_{1}(G[H])$*, and*$E_{N}M_{2}(G[H])=M_{2}(G[H])$*.*

Corollary 3.21 *If G has at least one vertex with*ε(u)=1*and H does not have any vertex with*ε(u)=1*, then*

$$E_{N}M_{1}(G[H])=4M_{1}(G[H]),\;E_{N}M_{2}(G[H])=4M_{2}(G[H]),$$

$$E_{N}M_{3}(G[H])=2M_{1}(G[H]).$$

Example 3.22 For n≥4, we have

$$E_{N}M_{1}(P_{2}[P_{n}])=8n^{3}+32n^{2}-48,$$

$$E_{N}M_{2}(P_{2}[P_{n}])=4n^{4}+24n^{3}+24n^{2}-48n-48,$$

$$E_{N}M_{3}(P_{2}[P_{n}])=4n^{3}+16n^{2}-24.$$

### 3.6 Symmetric difference

The symmetric deference [11] G⊕H is defined by V(G⊕H)=V(G)×V(H) and E(G⊕H)={((a,b),(c,d)):ac∈E(G)orbd∈E(H)butnotboth}.

Lemma 3.23 *For any two graphs G and H*

$$\delta_{en_{G⊕H}}(u,v)=2d_{G⊕H}(u,v)$$

Theorem 3.24 *For any two graphs G and H*

$$E_{N}M_{1}(G⊕H)=4M_{1}(G⊕H),$$

$$E_{N}M_{2}(G⊕H)=4M_{2}(G⊕H),$$

$$E_{N}M_{3}(G⊕H)=2M_{1}(G⊕H).$$

Example 3.25 $$E_{N}M_{3}(P_{2}⊕P_{n})=4n^{3},$$

$$E_{N}M_{1}(P_{2}⊕P_{n})=2E_{N}M_{3}(P_{2}⊕P_{n}),$$

$$E_{N}M_{2}(P_{2}⊕P_{n})=nE_{N}M_{3}(P_{2}⊕P_{n}).$$

## 4 Conclusion

In this article, we've introduced new indices referred to as eccentric neighborhood Zagreb indices. These indices have been conceptualized and their discriminating power investigated with regard to the predictability of the boiling point of the chemical substances, as the correlation coefficients between 0.9814 and 0.993 were acquired greater than the ones received in the case of eccentric connectivity and Winner indices. We have calculated those indices for some graphs and additionally studied a number of their characteristics. We've got calculated the formulation for some graph operations such as join, disjunction, composition, and symmetric deference. Because these indices are appearing for the first time, we have some issues to address in the feature, such as

1. Define some new versions of these topological indices that are parallel to the usual Zagreb topological indices.
2. Which graphs have the highest value for those indices as well as the lowest value?
3. The investigation of bounds is still an open area to study.
4. The mathematical relationships between the new and prior indices.
5. Used such indices to analyze certain significant chemical substances.
6. Investigating the polynomials that are connected to these indices.

## CRediT authorship contribution statement

**Hanan Ahmed:** Performed the experiments; Analyzed and interpreted the data; Wrote the paper.

**Anwer Saleh:** Conceived and designed the experiments.

**Rashad Ismail, Ruby Salestina M, Abdu Alameri:** Contributed reagents, materials, analysis tools or data.

## Declaration of Competing Interest

The authors declare that they have no competing interests.

## Data Availability

Data included in article/supp.material/referenced in article
